# Planar cell polarity: intracellular asymmetry and supracellular gradients of Frizzled

**DOI:** 10.1098/rsob.230105

**Published:** 2023-06-14

**Authors:** José Casal, Freya Storer, Peter A. Lawrence

**Affiliations:** Department of Zoology, University of Cambridge, Downing Street, Cambridge CB2 3EJ, UK

**Keywords:** Frizzled, gradients, planar cell polarity

## Abstract

Planar cell polarity (PCP), the coordinated orientation of structures such as cilia, mammalian hairs or insect bristles, depends on at least two molecular systems. We have argued that these two systems use similar mechanisms; each depending on a supracellular gradient of concentration that spans a field of cells. In a linked paper, we studied the Dachsous/Fat system. We found a graded distribution of Dachsous *in vivo* in a segment of the pupal epidermis in the abdomen of *Drosophila*. Here we report a similar study of the key molecule for the Starry Night/Frizzled or ‘core’ system. We measure the distribution of the receptor Frizzled on the cell membranes of all cells of one segment in the living pupal abdomen of *Drosophila*. We find a supracellular gradient that falls about 17% in concentration from the front to the rear of the segment. We present some evidence that the gradient then resets in the most anterior cells of the next segment back. We find an intracellular asymmetry in all the cells, the posterior membrane of each cell carrying about 22% more Frizzled than the anterior membrane. These direct molecular measurements add to earlier evidence that the two systems of PCP act independently.

## Graphical abstract

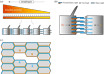

## 1. Introduction

**Figure 1.**
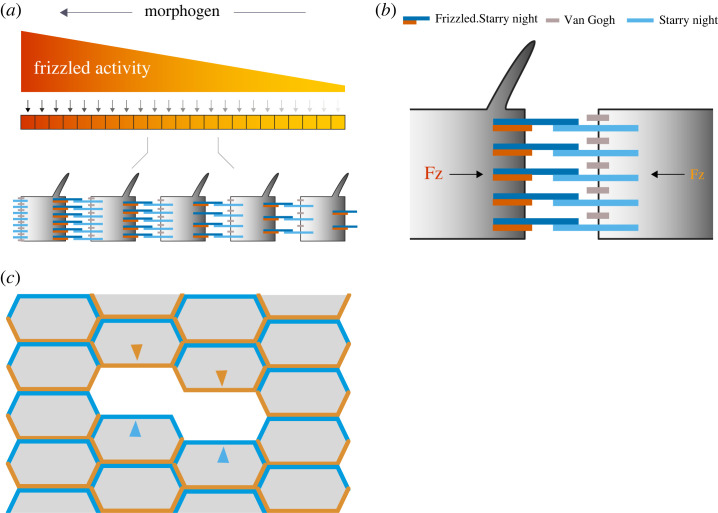
Model of the Stan/Fz system. (*a*,*b*) A whole segment in the abdomen is shown. In response to gradient(s) of morphogen(s), a supracellular gradient of Fz activity is established. This gradient may result from regulated transcription of *frizzled* itself and/or of one or more of those components of the Stan/Fz system that can influence the activity of Fz. The heart of the system consists of Fz.Stan dimers in the membrane of one cell interacting with Stan molecules in the membrane of a neighbour cell. Vang, located in the neighbour cell, promotes this interaction. Each cell determines its polarity by comparing the levels of Fz.Stan and Stan between its anterior and posterior membranes [14]. (*c*) How we measure tagged Fz in anterior and posterior membranes. All the cells contain normal amounts of Fz, half of which is tagged (shown as colour in each cell's membrane). All the tagged Fz is removed in small clones (a two-cell clone is shown) and replaced with normal untagged Fz. Thus, we can measure the tagged Fz that belongs to the posterior membrane (orange) or to the anterior membrane (blue) of a cell neighbouring the clone.

This short article is supplementary to Chorro *et al*. [1] but presents new and important results. For an introduction and the historical background, we refer readers to that paper [1]. Briefly, the topic is planar cell polarity (PCP) and its relationship to supracellular gradients [2–4]. In *Drosophila* two independent molecular systems build PCP (reviewed in [5]), and each system is thought by some to rely on a supracellular molecular gradient, its orientation defining and coordinating the polarities of individual cells [5,6]. The Dachsous/Fat (Ds/Ft) system depends on gradients of the cadherin Dachsous and the Golgi kinase Four-jointed; in the abdomen both align with the anteroposterior axis. In each segment there are two opposing gradients of the Ds protein: in the anterior (A) compartment the Ds gradient rises from anterior to posterior but falls again in the posterior (P) compartment [7] (reviewed in [5,8]). For Dachsous, measurements were made *in vivo* of the intracellular asymmetry in each cell. Over an entire segment both supracellular gradients were plotted with respect to the organizing compartment borders and it was found that the gradient of the P compartment bleeds slightly into the A compartment [1]. Now we present results with the second system, known as the ‘core’ or the Starry Night/Frizzled (Stan/Fz) system [9–12]. To determine its polarity a cell compares the levels of Fz activity of its neighbours and points its hairs and bristles towards its lowest neighbour. We proposed that a supracellular gradient of activity of Fz coordinates the polarity of many cells [13] (figure 1). In a comparable way to the Ds/Ft system, Stan/Fz uses asymmetric molecular bridges that link neighbouring cells. Each bridge consists of a dimer of two Stan cadherin molecules, one in each cell and only one of which is bound to an Fz molecule. The orientations and placements of Stan-Stan.Fz and Fz.Stan-Stan bridges (the hyphen denotes the interface between two cells) in the anterior and posterior membranes of individual cells stem from a supracellular gradient of Fz activity [5,14] (reviewed in [8]). Here we measure and describe the intracellular and supracellular distribution of the Fz molecule, *in vivo*, in the developing adult abdomen of *Drosophila*.

Our main findings are:

(i) There is a strong intracellular asymmetry in the location of Fz in cells over the entire segment. There is about 22% difference in Fz levels between the posterior and anterior membranes of each cell.
(ii) There is a shallow and monotonic gradient of Fz amount. It is near flat in the most anterior part of the segment but the amount of Fz steadily declines from there to reach a minimum at the posterior limit of the P compartment, encompassing about 17% difference in levels.
(iii) The amounts of Fz climb upwards in the most anterior cells of the next segment back to reach the same scalar value found at the front of the previous segment. Thus, the gradient is reset in each segment.

## 2. Results

### 2.1. Distribution across the metamere

**Figure 2.**
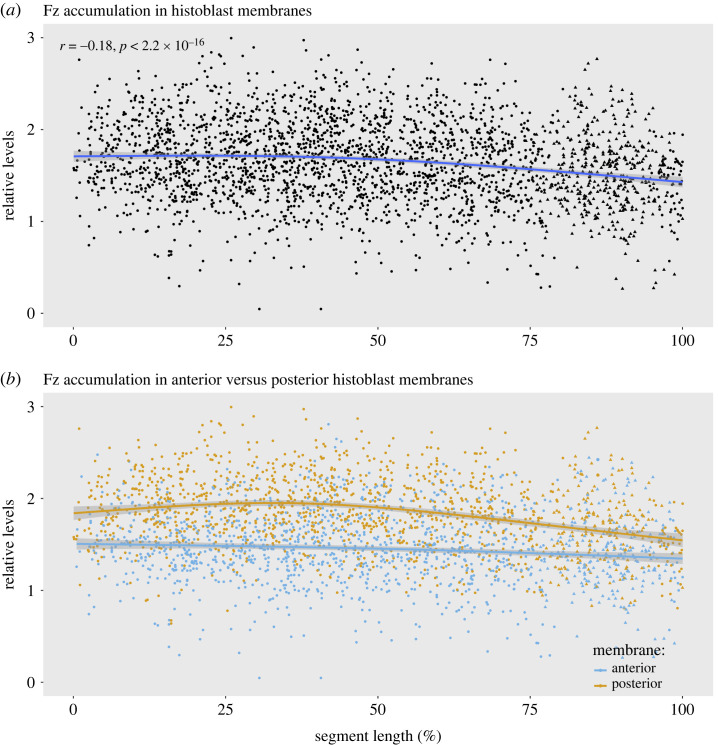
The supracellular gradient and cellular asymmetry of Fz in wild-type pupal epidermis. (*a*) Smoothed conditional means plots. All the individual measurements we made of Fz (both anterior and posterior cell membranes) are plotted across an entire metamere (0–100% of segment length). Measurements of cells of the A and P compartments are shown as black circles and triangles, respectively. P compartment cells were identified because they expressed *engrailed*. The Pearson correlation coefficient was calculated. Supracellular gradients fall from the front to the back of the segment. There is a difference of 17% in relative levels in the segment (where *a* is the anterior limit and *b* the posterior limit of the gradient (*percentage difference =* |*(a−b)* |*/((a + b)/2) × 100*). The shaded area encompasses the 95% confidence interval for the fitted curve. (*b*) The data points from (*a*) are shown separately as deriving from anterior (blue) or from posterior cell membranes (orange). Note both sets of data are graded but differ consistently in the relative Fz levels. The amount of Fz at the anterior membranes declines evenly from the front to the back, whereas the amount of Fz at the posterior membranes appears to peak near the middle of the A compartment. The difference in relative levels of Fz between anterior and posterior membranes across the whole segment including A and P compartments is 22.5% ± 0.8 (mean and 95% CI), ranging from 16.6 to 27.3%.

Each segment comprises one anterior (A) and one posterior (P) compartment; we mark the P compartment so all its cells are labelled [1]. We measure the intensity of fluorescence due to molecularly tagged Fz on single anterior and posterior membranes of many individual diploid cells (adult histoblasts) over a whole abdominal segment of the living pupa. A total of 2440 data points were taken from single-cell membranes, and these were plotted with respect to position in the anteroposterior axis of the segment (figure 2*a*). Data points from the anterior and posterior membranes were then separated and plotted as two sets, each with respect to position in the anteroposterior axis (figure 2*b*).

Note that the A compartment is divided into two distinct subdomains [15,16], the anterior domain (roughly, 0–25% in figure 2*a*) being about half the length of the posterior. We find a gentle gradient in the amount of Fz protein, falling from anterior to posterior of the A compartment as a whole (correlation coefficient *r* = −0.18). A region corresponding approximately to the anterior subdomain shows a flat distribution of Fz that steepens to fall in the remainder of the segment, including the P compartment. An additional analysis of the intersegmental region with more data points suggests that, within the anteriormost cells of the A compartment, the level returns to that typical of the top of the segmental gradient (electronic supplementary material, figure S1b). We imagine this rise to be abrupt, perhaps occurring only over one or two cells (mirroring the distribution of Dachs which shifts from the back of the cell to the front over about two cells [1]).

### 2.2. Cellular asymmetry

Within each epithelial cell, there is strong asymmetry in the distribution of Fz. In the developing wing, there is more Fz at the distal side of the cell [17]. In the pupal abdomen and as was predicted [14], there should be more Fz on that side of the cell facing towards the lower level of the gradient. Figure 2*b* and electronic supplementary material, figure S2 show that indeed this is true across the whole segment. Figure 2*b* also shows that the anterior and posterior membranes behave differently; the amount of Fz in the anterior membranes declines slightly but steadily from the front to the back, while the amount of Fz in the posterior membranes shows a gentle rise in the front part of the segment and a steeper fall in the back.

## 3. Discussion

PCP is a field in flux. Even within flies there are several anatomical regions (wing, abdomen and eye) that are being studied and, for each region, various models of mechanism have been advanced. Here we emphasize and simplify the two main schools of thought. In one, the Stan/Fz system is believed to drive PCP autonomously by means of a gradient of Fz activity. Thus, experiments with small clones had argued that hairs point from cells expressing more Fz to cells expressing less, leading to this view of the fly abdominal segment: ‘all cells make hairs and bristles that point posteriorly, suggesting there is a continuous gradient of Fz activity from high to low, from anterior to posterior’ [13]. The other school holds that the Stan/Fz (the ‘core’) system cannot orient PCP on its own because it is oriented by the Ds/Ft system, possibly in concert with other pervasive signals: ‘The core module has no apparent intrinsic mechanism for orienting its action to the tissue axes’ [18].

In order to help adjudicate between these two viewpoints, we study the distribution of the Fz molecule directly. We quantify the amount of Fz protein in all cell membranes of an abdominal segment in the living pupa. We find an overall gradient that falls from the front of the segment to the back and repeats in the next segment. Because the relationship between Fz amount and Fz activity is not straightforward (see below), our measurements suggest but do not prove a gradient of Fz activity. A supracellular gradient of Fz activity has been proposed, modelled and discussed elsewhere [13,14,19,20] but not its relationship to amount. The actual amounts found are themselves subject to the methods used, the nature of the label and the sensitivity of the recording devices. We therefore believe that our most significant findings are the existence of a gradient per se, its direction of decline and its range, as well as the asymmetric distribution of Fz. Even so we cannot claim with certainty that the gradient and the cell asymmetry we observe are the causes and not the consequences of PCP.

Strutt observed long ago that, in wing cells, Fz is concentrated in the distal membrane [17] and Vang in the proximal membrane [21], and these complementary distributions have been confirmed by others (reviewed in [22]). Vang accumulates at the anterior membranes of abdominal cells [23], and we confirm now that Fz is concentrated in the posterior membranes of these cells. Importantly, we find that all cells of the metamere show similar degrees of asymmetry.

The shape of the gradient may be important, particularly a flat section at the front of the A compartment. One might expect that the flat section would correlate with a region of reduced cellular asymmetry for Fz, but this is not the case, arguing against any simple relationship between Fz amount and Fz activity. Other components of the Stan/Fz system, such as Van Gogh (Vang), Prickle or Dishevelled, could also be distributed in a graded manner and contribute to a Fz activity gradient.

We know from many early experiments using small clones, that interfaces between cells carrying different amounts of Fz can drive cell polarity [13,24–27]. Here we have demonstrated a gradient of Fz amount, and this could result from gene expression or from graded activity of the Stan/Fz system [13]. This gradient is likely to act in a complex way. Earlier we presented evidence that the Hedgehog (Hh) morphogen, which is produced by cells of the P compartment, enters the A compartment from both the front and the back. We showed it acts separately on the Ds/Ft and Stan/Fz systems [28]. Hh may regulate the Stan/Fz system via Fz, but it may also affect the distribution of Vang, Prickled or Dishevelled.

How might Hh regulate Fz? Hh is thought to activate Decapentaplegic and Wingless expression in the most posterior cells of the A compartment. These proteins could therefore peak at the back of A, forming gradients going forward into A and backwards into the P compartment. If these gradients were used to drive Fz activity and thereby PCP, they would need to do so with opposite sign in the A and P compartments (because hairs point backwards in both compartments). Such rectification might be achieved via the deployment of the Prickle gene that acts on both PCP systems [29]. There could even be an undiscovered morphogen, driven by Hh entering from the anterior, that runs from the front of A to the back of P and, if so, could drive Fz activity more simply.

These findings relate to long standing and disparate views on how the two systems of PCP, the Ds/Ft and the core or Stan/Fz systems, work together to polarize cells. A three-tier model was proposed [30]: at first, the Ds/Ft system is imagined to act over a large field to provide global directional information. Second, this directional signal, weak in itself, is amplified within the cell to provide a strong asymmetry of the Stan/Fz system and the localization of its proteins, thereby establishing PCP in each cell and coordinating it in many cells. Finally, a third set of molecules translates these asymmetries in each cell into polarized morphology. This three-tier hypothesis was challenged by evidence from the abdomen that the Ds/Ft system can function to polarize the cells in the absence of the Stan/Fz system [28]. This and other experiments led to the conclusion that the two systems work independently and can even conflict with each other [31], a conclusion that was widely (discussed in [6]), but not universally accepted [18]. The Fz gradient described here does not easily support the three-tier model because the Ds (Ds/Ft system) and Fz gradients (Stan/Fz system) have completely different topographies. Also, any feedback amplification would tend to level out small differences between cells and make those differences more difficult to detect.

However and even so, there was an attractive hypothesis as to how the Ds/Ft and Stan/Fz systems might be linked: it was suggested that microtubules could be oriented by Ds/Ft and thereby polarize the intracellular transport of molecules such as Fz [32,33]. But some key published data were questioned when reexamined and the model challenged by new observations on microtubules in polarized larval and adult abdominal cells [34]. Microtubule organization is nevertheless related to cell shape in several different kinds of epidermis [34,35].

Finally, an important question remains unanswered: how does the cell compare the disposition of PCP proteins in its anterior and posterior membranes in order to read its polarity? With respect to the Ds/Ft system, it was found that when one membrane of a single cell abuts two different neighbours, parts of that cell may develop two opposing polarities [36]. To explain it was suggested the cell uses oriented ‘conduits’ to allow and yet spatially restrict the comparison between opposing membranes. It is not known whether this hypothesis could apply to the Stan/Fz system and, even if it does, how a comparison of the amounts and orientations of Stan-Stan.Fz and Fz.Stan-Stan in opposing membranes might be made.

### 3.1. Opening up

PCP is much more than we have worked on; we see it as a huge unsolved problem in Developmental Biology. Every cell in an embryo needs access to information of its identity (its germ layer, its provenance, its combination of transcription factors), its position (is it in the outer or inner cell layer, where is it in terms of the anteroposterior or dorsoventral axis) and its polarity (in which direction shall it act with other cells to build pattern, to move, to divide, to extend an axon or orient a cilium?). Much work has been done on the first two problems and relatively little on the last. Most progress with PCP has come from research on *Drosophila*, in which a combination of a century of genetics, sophisticated genetic mosaics and logic has yielded understanding of fundamental mechanisms. The two molecular systems, the Ds/Ft and Stan/Fz systems discovered in flies also operate in vertebrates [6]. Knock out both these systems in *Drosophila* and the result is an adult insect that has poorly oriented hairs and bristles but nevertheless has gone through most of development to build an almost-fly with normal structures. Thus, we believe that even if we were to understand these two systems properly, which we do not, we would still lack a complete picture of PCP.

We have worked on and off in the field of PCP since 1962 and our time has run out. During this period, we have seen PCP wax and wane in fashion and now few experts remain and we fear the field may become overlooked. We in PCP have also been guilty of obscurantism and factionalism, neither of which has helped the clarity of our papers. Also, modern science practice and metrics disfavour small fields; an area of research needs a certain size to build momentum and sell itself. Consequently, biology can become dominated by a few fashionable and overcrowded areas of study. We therefore appeal to the scientific community to support those who research PCP, especially on *Drosophila*, and for some to join them.

## 4. Material and methods

### 4.1. General note

The methods used here are comparable to those used to plot the distribution of Ds over the pupal segment [1]. A summary of this approach with Fz is shown in figure 1. We find that Fz is distributed in a completely different pattern to that of Ds, both over the whole segment and in its intracellular asymmetry. This contrast with Ds is important because it eliminates a concern raised by a reviewer of Chorro *et al*. [1] who asked whether our findings with Ds could be due to variables in the background (such as a systematic variation in fluorescence absorption by the cuticle). Note, also, that our findings for both Fz and Ds both fit with much earlier and separate experiments predicting that the two proteins would be distributed differently and indeed would be as we now report them, both within the segment and in the cell [7,28].

We plot relative values of fluorescence (the signal relative to a standard measured at the back of A compartment). The exact numbers are of less interest than the relative numbers as the exact numbers may be influenced by extrinsic factors such as the quality of the sensors and the fluorescence intensities of the tags.

### 4.2. Mutations and transgenes

Flies were reared at 25°C on standard food. The FlyBase [37] entries for the mutant alleles and transgenes used in this work are the following:

*hs.FLP: Scer\FLP1^hs.PS^; en.Gal4: Scer\GAL4^en-e16E^; UAS.DsRed: Disc\RFP^UAS.cKa^; Df(3L)GN50; fz::GFP: fz^EGFP.C^*

### 4.3. Experimental genotype

*y w hs.FLP/ +; en.Gal4 UAS.DsRed/ +; Df(3L)GN50 fz::GFP FRT80/ FRT80*

### 4.4. Live imaging and quantification of Frizzled

To induce clones expressing untagged *fz* clones we followed the protocol of Chorro *et al*. [1]. Briefly, pupae of the appropriate genotype were heat shocked at 24 h after puparium formation. Twenty-four hours later the pupae were removed from the puparium and examined using a Leica SP5 inverted confocal microscope with a 63x NA 1.4 oil immersion objective. Z-stacks of images of 1024 × 1024 pixels covering the whole A and P compartments of a segment were acquired. The stacks were opened in Fiji and converted to single images with the Maximum Intensity Projection algorithm. The coordinates of the compartment borders were obtained, as well as the coordinates and fluorescence intensity of membranes at clone borders. The fluorescence intensities were standardized with respect to the intensity of a region free of clones abutting the A/P border. The ***relative levels*** used in the plots were calculated as ***log(relative intensity) – 3*.** Percentage difference of Fz accumulation between compartment borders or between anterior and posterior membranes was calculated using the formula ***percentage difference =* |*(a−b)* | */ ((a + b)/2) × 100***, where *a* and *b* are *relative levels*.

### 4.5. Statistics and plotting

We used RStudio with R v.4.1.2 [38], and the *tidyverse* [39] and *mgcv* [40] packages.

## Data Availability

Data used in figure 2 and electronic supplementary material, figures S1 and S2 can be obtained from the University of Cambridge Open Access repository: https://doi.org/10.17863/CAM.96331 [41]. Supplementary material is available online [42].
